# Co-targeting CD47 and VEGF elicited potent anti-tumor effects in gastric cancer

**DOI:** 10.1007/s00262-024-03667-9

**Published:** 2024-03-27

**Authors:** Kaiqi Zhang, Yuan Xu, Xusheng Chang, Caili Xu, Wenjing Xue, Dan Ding, Mingming Nie, Hui Cai, Jun Xu, Lu Zhan, Jiangbo Han, Tiancai Cai, Dianwen Ju, Li Feng, Xuyao Zhang, Kai Yin

**Affiliations:** 1 Department of Gastrointestinal Surgery, Changhai Hospital, Naval Medical University, Shanghai, 200433 China; 2 Department of Biological Medicines & Shanghai Engineering Research Center of Immunotherapeutics, School of Pharmacy, Fudan University, Shanghai, 201203 China; 3 Department of Sanatorium and Nursing Section, Xiamen Special Service Health Center, Xiamen, 361005 China; 4 Department of Endoscopy Center, Minhang Hospital, Fudan University, 170 Xinsong Road, Shanghai, 201199 China

**Keywords:** Anti-CD47 therapy, Innate immunity, Angiogenic vasculature, VEGF, Combinational therapy

## Abstract

**Background:**

CD47, serving as an intrinsic immune checkpoint, has demonstrated efficacy as an anti-tumor target in hematologic malignancies. Nevertheless, the clinical relevance of CD47 in gastric cancer and its potential as a therapeutic target remains unclear.

**Methods:**

The expression of CD47 in clinical gastric cancer tissues was assessed using immunohistochemistry and Western blot. Patient-derived cells were obtained from gastric cancer tissues and co-cultured with macrophages derived from human peripheral blood mononuclear cells. Flow cytometry analyses were employed to evaluate the rate of phagocytosis. Humanized patient-derived xenografts (Hu-PDXs) models were established to assess the efficacy of anti-CD47 immunotherapy or the combination of anti-CD47 and anti-VEGF therapy in treating gastric cancer. The infiltrated immune cells in the xenograft were analyzed by immunohistochemistry.

**Results:**

In this study, we have substantiated the high expression of CD47 in gastric cancer tissues, establishing a strong association with unfavorable prognosis. Through the utilization of SIRPα-Fc to target CD47, we have effectively enhanced macrophage phagocytosis of PDCs in vitro and impeded the growth of Hu-PDXs. It is noteworthy that anti-CD47 immunotherapy has been observed to sustain tumor angiogenic vasculature, with a positive correlation between the expression of VEGF and CD47 in gastric cancer. Furthermore, the successful implementation of anti-angiogenic treatment has further augmented the anti-tumor efficacy of anti-CD47 therapy. In addition, the potent suppression of tumor growth, prevention of cancer recurrence after surgery, and significant prolongation of overall survival in Hu-PDX models can be achieved through the simultaneous targeting of CD47 and VEGF using the bispecific fusion protein SIRPα-VEGFR1 or by combining the two single-targeted agents.

**Conclusions:**

Our preclinical studies collectively offer substantiation that CD47 holds promise as a prospective target for gastric cancer, while also highlighting the potential of anti-angiogenic therapy to enhance tumor responsiveness to anti-CD47 immunotherapy.

**Supplementary Information:**

The online version contains supplementary material available at 10.1007/s00262-024-03667-9.

## Introduction

Gastric cancer, being one of the most widespread and lethal forms of cancer, constitutes 5.6% of newly diagnosed cancer cases and 7.7% of cancer-related fatalities on a global scale [1]. As a consequence of being frequently diagnosed at an advanced stage, patients often fail to seize the optimal surgical intervention window, resulting in a significant mortality rate [2]. In recent times, there has been a growing trend, wherein a greater proportion of patients diagnosed with gastric cancer have been deriving advantages from immunotherapies. Nevertheless, the majority of immunotherapeutic interventions that have obtained approval for clinical utilization primarily concentrate on activating the adaptive immune system, thereby overlooking the crucial contribution of the innate immune system in the context of cancer therapy [3].

CD47, which has been recognized as an intrinsic immune checkpoint, exhibits extensive overexpression in diverse types of tumor cells [4–6]. The interaction between CD47 and signal regulatory protein alpha (SIRPα) directly hinders the phagocytosis of tumor cells by macrophages, leading to a subsequent impairment in the presentation of tumor antigens to T lymphocytes [7–9]. CD47 exhibits a strong association with unfavorable prognosis in patients diagnosed with non-small-cell lung cancer, melanoma, and acute myeloid leukemia [10–12]. The augmentation of macrophage phagocytosis of tumor cells has been observed in various preclinical tumor models, such as small-cell lung cancer, anaplastic thyroid carcinoma, and glioblastoma, through the implementation of monoclonal antibodies or fusion proteins to obstruct the CD47/SIRPα axis [13–15]. However, the clinical significance of CD47 expression in gastric cancer and the efficacy of targeting CD47 as a strategy for eradicating tumor cells have yet to be fully understood.

Although immune checkpoint inhibitors (ICIs) have demonstrated significant efficacy in various cancers, their benefits are limited to a small subset of patients, and their effectiveness is constrained in clinical settings. Tumors can evade immune system responses by establishing an immunosuppressive tumor microenvironment (TME) via local angiogenesis [16–18]. Vascular endothelial growth factor (VEGF) and its receptor (VEGFR) exhibit excessive activity in various tumor types, thereby stimulating endothelial cell proliferation and facilitating microangiogenesis [19–21]. The aberrant formation of new blood vessels exacerbates the oxygen deficiency and acidic conditions within the TME, thereby facilitating additional blood vessel growth and immune system suppression [22–24]. An elevated concentration of VEGF hampers the maturation process of dendritic cells, and impedes T-cell infiltration and cytotoxic activity, while concurrently promoting the recruitment and proliferation of pro-tumor M2-like macrophages [25–28]. The administration of appropriate doses of VEGF inhibitor facilitates vascular normalization and transforms the immunosuppressive tumor microenvironment (TME) into an immunosupportive milieu [23]. The evaluation of combining anti-angiogenic agents and anti-PD-L1 ICIs has been conducted as a result of the influence of anti-angiogenesis therapy on the tumor immune microenvironment. It has been reported that anti-angiogenic therapy enhances the efficacy of anti-PD-L1 therapy in pancreatic neuroendocrine tumors by promoting the development of intratumoral high endothelial venules, which in turn facilitates increased infiltration and activity of cytotoxic T cells [29]. In the context of gastric cancer, anti-angiogenic therapies have demonstrated efficacy, leading to the approval of ramucirumab for the treatment of advanced gastric cancer [30]. Ongoing clinical trials are currently investigating the efficacy of combining VEGF/VEGFR blockade with PD-1/PD-L1 inhibitors, such as mepolizumab, pembrolizumab, and durvalumab, for the treatment of solid tumors, including gastric cancer [31]. Nevertheless, there remains a lingering uncertainty regarding the role of angiogenesis in the application of innate immune checkpoint blockade therapy.

In this context, a comprehensive analysis was conducted on a cohort of 89 patients diagnosed with gastric cancer, to elucidate the clinical relevance of CD47 in the context of gastric cancer. Subsequently, patient-derived cell (PDC) models and humanized patient-derived xenograft (Hu-PDX) models were utilized to evaluate the therapeutic efficacy of anti-CD47 immunotherapy in suppressing tumor growth. Additionally, the impact of anti-CD47 immunotherapy on the microvessel network within the TME was investigated, along with the potential correlation between anti-CD47 immunotherapy and anti-angiogenic therapy. Our study has shown that CD47 serves as a viable therapeutic target for gastric cancer, and it has also presented a potentially effective combination approach involving the inhibition of both the CD47/SIRPα axis and angiogenesis for the treatment of this disease.

## Materials and methods

### Reagents and antibodies

Anti-CD80 monoclonal antibody (66,406-1-Ig, Proteintech), anti-CD163 monoclonal antibody (GB13340, Servicebio), anti-CD8 monoclonal antibody (GB12068, Servicebio), anti-CD31 monoclonal antibody (GB113151, Servicebio), VEGFA Monoclonal antibody (19,003-1-AP, Proteintech), anti-CD47 monoclonal antibody (ab218810, Abcam), carboxyfluorescein diacetate succinimidyl ester (CFDA SE) (C0051, Beyotime), PerCP anti-CD68 (333,813, BioLegend), PE anti-CD11b (101,208, BioLegend), granulocyte–macrophage colony-stimulating factor (GM-CSF) (C003, novoprotein), FITC-labeled anti-CD47 (CC2C6, BioLegend), Human Lymphocyte separation medium (7,111,011, DAKEWE). The fusion protein SIRPα-Fc has been engineered based on the initial extracellular domain of SIRPα and is currently undergoing Phase I/II clinical trial (NCT05140811) [32]. SIRPα-VEGFR1 is constructed by combining the extracellular domain of SIRPα with that of VEGFR1 (GenBank accession number: MG920788).

### Patients

Written informed consent was obtained from all patients according to the guidelines established by the Clinical Research Ethics Committee of Changhai Hospital, which approved the current study (CREC: CHEC-2021-119). The Department of Gastrointestinal Surgery at Changhai Hospital collected gastric cancer tissues and their corresponding adjacent tissues from a total of 89 patients between the years 2021 and 2022. None of the patients underwent pre-surgical radiotherapy. Clinicopathological characteristics were gathered, and the T classification, N classification, and TNM stage were determined following the American Joint Committee on Cancer (AJCC) TNM staging system.

### Immunohistochemical analysis

The specimens were prepared and processed per the provided instructions. Subsequently, the slides underwent staining using primary antibody, secondary antibody, DAB, and hematoxylin, respectively [33]. Pathologists assessed the expression abundance of CD47 and VEGF by evaluating the immunohistochemical (IHC) score for each field. This score was determined based on the intensity of cytoplasm and membrane staining (strong = 3, moderate = 2, weak = 1, no staining = 0) as well as the proportion of stained cells (76–100% = 4, 51–75% = 3, 26–50% = 2, 5–25% = 1, 0–5% = 0). The composite score, which ranged from 0 to 12, was calculated by multiplying the stained cell range score and the intensity score. The final score was determined by taking the average of the composite scores obtained from 5 representative fields.

### Bioinformatics analysis

The Kaplan–Meier plotter (KM-plotter, http//kmplot.com) was utilized to investigate the association between CD47 expression and the survival outcomes of patients with gastric cancer. Additionally, the Gene Expression Profiling Interactive Analysis (GEPIA, http://gepia.cancer-pku.cn) database was employed to examine the potential correlation between the expression of CD47 and VEGF in gastric cancer.

### Immunoblot

The gastric cancer tissues were homogenized and combined with lysis buffer. Subsequently, the protein was separated using Sodium dodecyl sulfate–polyacrylamide gel electrophoresis (SDS-PAGE) and transferred onto a PVDF membrane via electrical means. The membranes were then obstructed using 5% bovine serum albumin (BSA) and subjected to incubation with a specific primary antibody. On the subsequent day, the membrane was immersed in a solution containing a secondary antibody. Ultimately, the immunoreactive bands were observed to measure the protein content.

### Cell and flow cytometry

The gastric cancer cell lines PDC1 and PDC2 were isolated from freshly obtained gastric cancer tissues and subsequently cultured in RPMI1640 medium supplemented with 10% FBS (Gibco). To assess the expression of CD47, the cells were subjected to staining with FITC anti-CD47 antibody and subsequently analyzed using flow cytometry. Additionally, human peripheral blood mononuclear cells (PBMCs) were isolated from healthy donors and subjected to incubation with GM-CSF at a concentration of 50 ng/mL for a duration of 7 days, resulting in their differentiation into macrophages. Using flow cytometry, macrophages were identified as CD68^+^CD11b^+^ cells. Gastric cancer cells PDC1 and PDC2 were then labeled with carboxyfluorescein diacetate succinimidyl ester (CFDA SE) and co-cultured with macrophages in serum-free medium with or without SIRPα-Fc at 37 °C for 2 h. The ratio of tumor cells to macrophages was 2:1. The cells were subsequently incubated with PE anti-human CD11b antibody and analyzed using flow cytometry. The phagocytosis index represents the proportion of CD11b^+^CFDA SE^+^ macrophages among the total CD11b^+^ macrophages.

### Hu-PDX models

The mouse experiments were conducted in adherence to the protocols approved by the Animal Ethical Committee of the School of Pharmacy at Fudan University (AEC: 2020-12-SY-ZXY-01).

A strain of NOD/ShiltJGpt mice (NCG mice), which had been genetically modified to lack the Prkdc and Il2rg genes and were severely immunodeficient, were procured from GemPharmatech Co., Ltd. These mice (6–8 weeks old) were housed in a controlled environment free from pathogens.

To establish a Hu-PDX model, the NCG mice were subjected to intraperitoneal injection of Busulfan (25mg/kg) to suppress the bone marrow. After 24 h, human CD34^+^ hematopoietic stem cells, obtained from neonate cord blood using the CD34 Positive Selection Kit (17,897, STEMCELL Technologies), were administered to the NCG mice via the caudal vein. The reconstitution of the human immune system in the NCG mice was observed two weeks later. Subsequently, the gastric cancer patient-derived xenograft was implanted into the humanized NCG mice. Fresh gastric cancer tissues obtained from surgical procedures were aseptically divided into smaller fragments. NCG mice were anesthetized using 1–1.5% isoflurane. Subsequently, a 1-cm incision was made on the dorsal flank of the mice, and the tumor fragment was implanted. The implantation site was closed using subcutaneous sutures, and the wound was sterilized with iodine. Intraperitoneal injections of Signal regulatory protein α (SIRPα) -Fc (4.5 mg/kg), VEGFR1-Fc (3.5 mg/kg), SIRPα-Fc (4.5 mg/kg) + VEGFR1-Fc (3.5 mg/kg), and SIRPα-VEGFR1 (5mg/kg) were administered twice a week. In the gastric carcinoma recurrence model, fragments of fresh gastric cancer tissue were transplanted into NCG mice that had been humanized. Once the tumor volume reached 1000 mm^3^, the tumors were partially removed, with 1% of the original tumor mass remaining. Intraperitoneal injections of SIRPα-Fc (4.5 mg/kg) + VEGFR1-Fc (3.5 mg/kg) and VEGFR1-SIRPα (5 mg/kg) were administered twice a week for a duration of 2 weeks.

### Statistical analysis

The data analysis was performed using GraphPad Prism (version 6.01). The association between survival and the variables of interest was assessed using Kaplan–Meier curves. Statistical comparisons were conducted using a Student’s t-test or One-Way Analysis of Variance (ANOVA). The correlation between the two variables was evaluated using Pearson’s correlation test. A P value less than 0.05 was considered statistically significant.

## Results

### CD47 is overexpressed in gastric cancer and correlated with poor prognosis

**Fig. 1:**
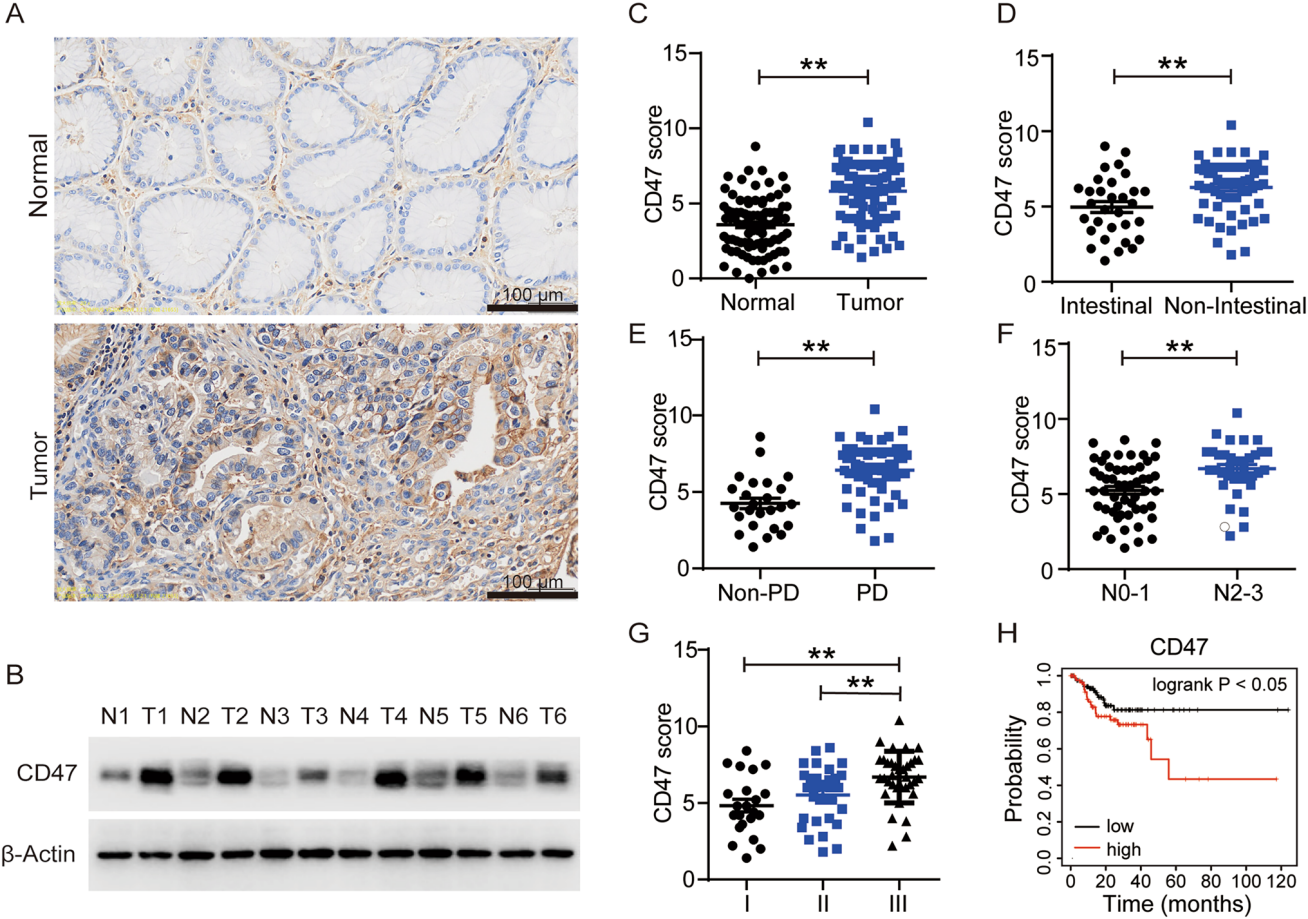
CD47 is overexpressed in gastric cancer tissues and is closely correlated with poor prognosis. **A** Representative images of immunohistochemical CD47 staining of normal mucosa and the tumor tissue of the stomach. **B** CD47 expression in normal mucosa and the tumor tissue of the stomach by immunoblot (N, normal; T, tumor). (**C**) The expression of CD47 in tumor and adjacent normal tissues of gastric cancer patients without classification. **D** The expression of CD47 in tumors of gastric cancer patients under the classification of intestinal and non-intestinal. **E** Non-poor differentiation and poor differentiation, **F** N0-1 and N2-3, and **G** TNM stage I, II, and III. **H** Relapse-free survival analysis conducted by Kaplan–Meier plotter according to CD47 expression of all patients. **P < 0.01

In light of the ongoing debate surrounding CD47 expression in gastric cancer, our study sought to assess the expression of CD47 in tumor tissues and their corresponding adjacent mucosae from a cohort of 89 gastric cancer patients. Immunohistochemical staining of both normal mucosae and tumor tissues revealed a significant upregulation of CD47 in gastric cancer tissues, irrespective of their differentiation stage (Fig. 1A, C). The verification of the finding was additionally supported by the immunoblot results (Fig. 1B). To investigate the association between CD47 expression and gastric cancer pathology (Supplemental Table S1), a further examination of the clinicopathologic variables was conducted, revealing elevated levels of CD47 expression in poorly differentiated and non-intestinal type gastric cancers, suggesting an unfavorable prognosis (Fig. 1D, E). Furthermore, the analysis demonstrated a significant association between CD47 expression and both lymph node metastasis and advanced tumor stage (Fig. 1F, G). Moreover, survival analysis conducted using the Kaplan–Meier plotter, which relied on RNA-seq data, further substantiated that elevated CD47 expression in individuals diagnosed with stomach adenocarcinoma generally indicated a diminished likelihood of relapse-free survival (Fig. 1H). These findings suggest that CD47 is extensively upregulated in gastric cancer tissues and exhibits a significant correlation with unfavorable prognosis.

### Targeting CD47 promoted macrophage phagocytosis of PDCs

**Fig. 2:**
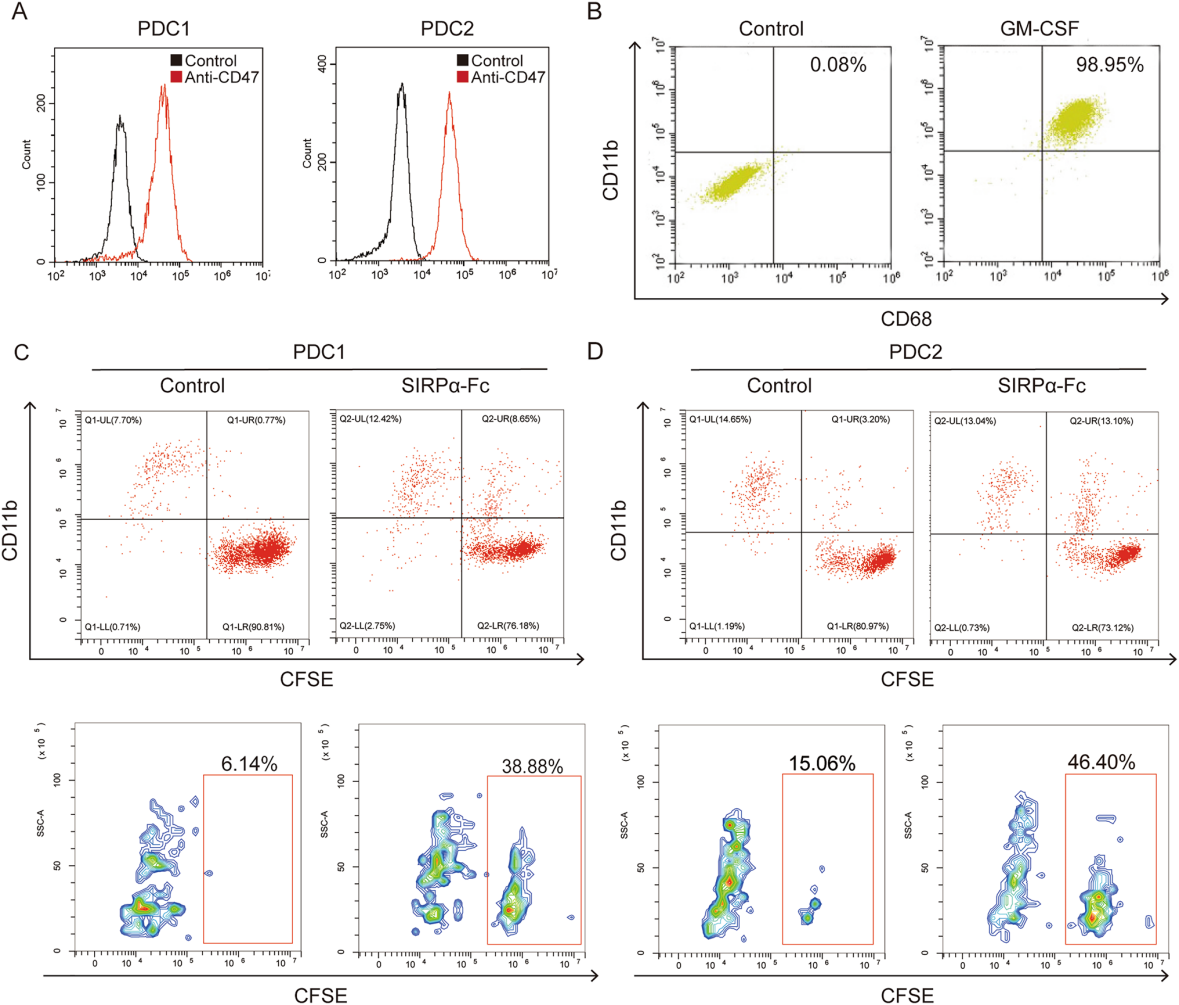
Targeting CD47 promotes macrophage phagocytosis of gastric cancer patient-derived cells. (**A**) FACS analysis for cell surface CD47 expression in gastric cancer patients-derived cells PDC1 and PDC2. **B** FACS analysis for CD68^+^CD11b^+^ macrophage derived from PBMC. **C**, **D** Representative flow cytometry plots depicting macrophage phagocytosis of PDC1 and PDC2 treated with SIRPα-Fc vs. control

Before examining the impact of targeting the CD47/SIRPα axis, we utilized CD47^+^ gastric cancer patient-derived cells (PDC1 and PDC2) and verified their identity through flow cytometry analysis (Fig. 2A). Subsequently, CFDA SE labeling was applied to PDC1 and PDC2, which were then co-cultured with macrophages derived from human peripheral blood mononuclear cells (Fig. 2B). The administration of SIRPα-Fc to co-cultured cells resulted in a notable augmentation in the phagocytic activity of macrophages toward PDC1 (from 6.14 to 38.88%) and PDC2 (from 15.06 to 46.40%) (Fig. 2C, D). These findings provide compelling evidence that the inhibition of CD47 can effectively enhance the phagocytic capability of macrophages toward gastric cancer cells.

### Targeting CD47 significantly inhibited the growth of gastric cancer

**Fig. 3:**
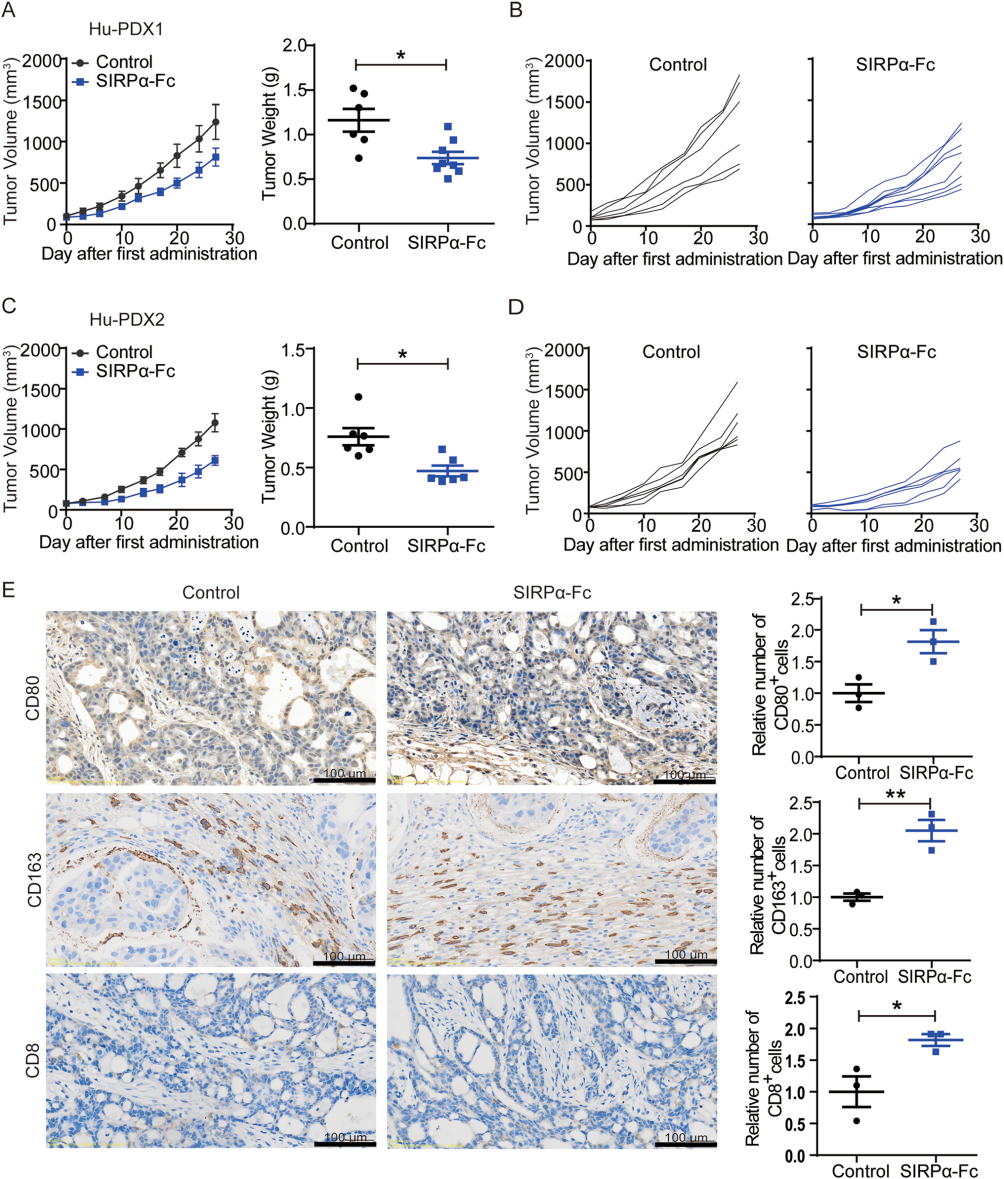
Targeting CD47 significantly inhibited the growth of gastric cancer in Hu-PDX models. **A** In the Hu-PDX1 model, tumor volume was measured twice a week and presented as mean ± SD. After treatment with SIRPα-Fc for 4 weeks, tumor weight was presented. (*n* = 6 for control group and *n* = 8 for SIRPα-Fc group, *P < 0.05, **P < 0.01) **B** Each line represented the tumor volume from an independent mouse in the Hu-PDX1 model. **C** In the Hu-PDX2 model, tumor volume was measured twice a week and presented as mean ± SD. After treatment with SIRPα-Fc for 4 weeks, tumor weight was presented. (*n* = 6 per group, *P < 0.05, **P < 0.01). **D** Each line represented the tumor volume from an independent mouse in the Hu-PDX2 model. **E** Representative photographs of immunohistochemical staining for CD80, CD163, and CD8 of tumor tissue sections and the number of CD80^+^, CD163^+^, and CD8^+^ cells in each group were normalized to the control group. The value of control was set to 1.0. (^*^P < 0.05, ^**^P < 0.01)

To accurately and authentically forecast the efficacy of anti-CD47 immunotherapy on human gastric cancer, we developed Hu-PDX models that maintained the attributes of the original tumors and provided a more accurate representation of the pertinent constituents and interactions within the TME. Mice-bearing tumors were randomly assigned to specified groups and treated with either SIRPα-Fc fusion protein or isotype control IgG1-Fc twice weekly for a duration of 4 weeks. The findings indicated that the group treated with SIRPα-Fc exhibited reduced tumor volume and weight compared to the control group, as depicted in Fig. 3A–D. Immunohistochemical analysis revealed an increase in both CD80^+^ M1 macrophages and CD8^+^ T cells infiltrating the TME following anti-CD47 therapy. However, it is important to note that there was also an increase in pro-tumor CD163^+^ M2 macrophages in the anti-CD47 treatment group (Fig. 3E). The findings suggest that the inhibition of CD47 resulted in an anti-tumor response by promoting the polarization of M1 macrophages and enhancing macrophage phagocytosis. However, it was also observed that CD47 blockade facilitated the infiltration of M2 macrophages.

### CD47 is positively correlated with VEGF in gastric cancer

**Fig. 4:**
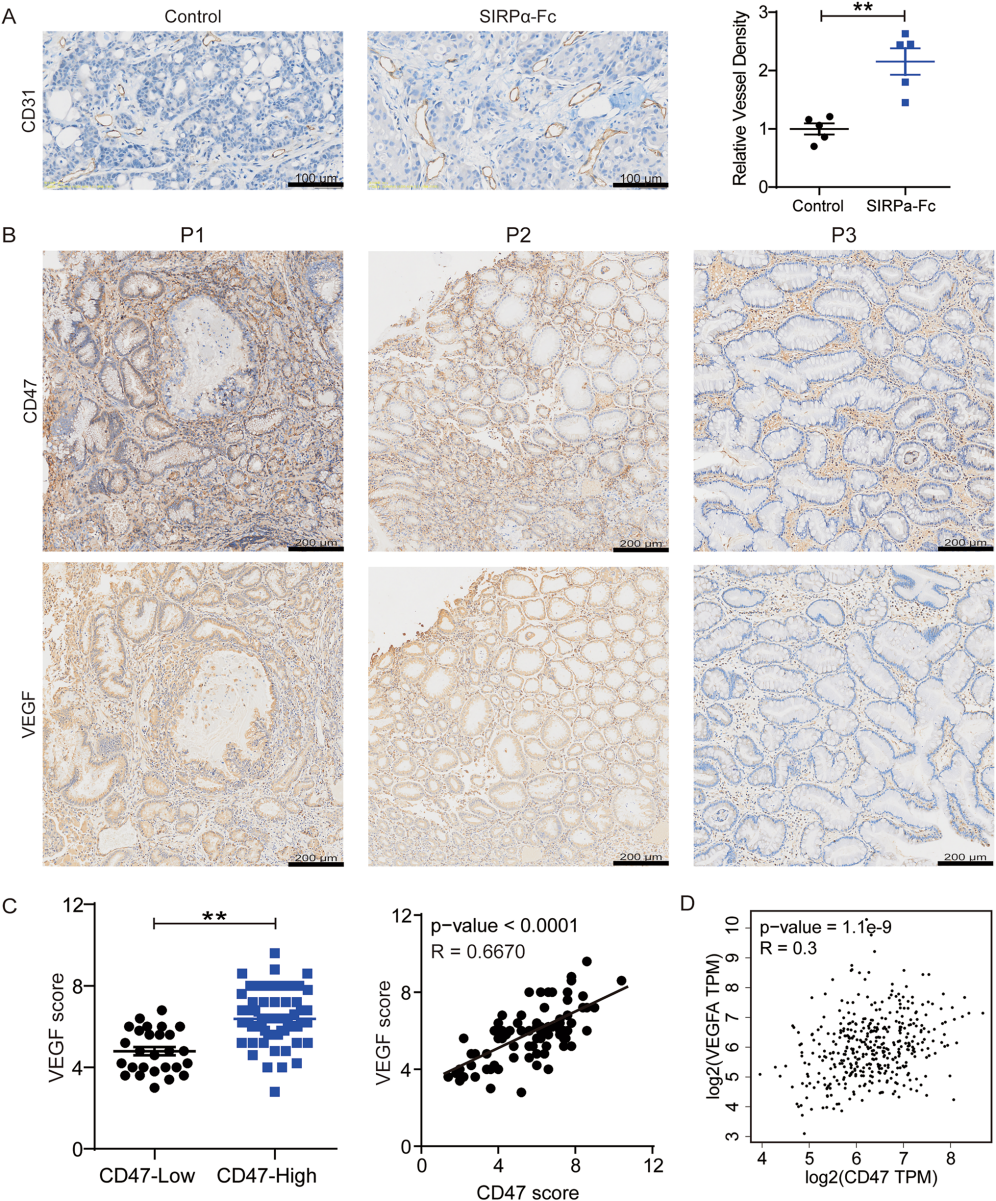
CD47 expression is positively correlated with VEGF expression in gastric cancer specimens. **A** Representative photographs of immunohistochemical staining for CD31 of tumor tissue sections and the relative vessel density in each group were normalized to the control group. The value of control was set to 1.0. (*P < 0.05, **P < 0.01). **B** Representative photograph of immunohistochemical staining for CD47 and VEGF of human gastric cancer tissue sections. **C** The VEGF expression in tumors of 89 gastric cancer patients under the classification of CD47 high and CD47 low and analysis of the correlation between CD47 and VEGF in the gastric cancer tissues.** D** Analysis of correlation between CD47 and VEGF in gastric cancer using the GEPIA database. *P < 0.05, **P < 0.01

Given the presence of an immunosuppressive tumor microenvironment in the context of CD47 blockade, it is imperative to elucidate the underlying mechanism and devise a strategy to enhance the efficacy of anti-CD47 therapy. The development of sustained tumor angiogenic vasculature frequently leads to the establishment of an immunosuppressive microenvironment. In this study, we conducted an analysis of intratumoral microvessel density within the TME and observed a significant increase in microvessel density following treatment with SIRPα-Fc (Fig. 4A). In addition, an examination was conducted on the relationship between VEGF and CD47 in gastric cancer. Notably, an analysis of samples obtained from patients with gastric cancer using immunohistochemistry revealed a novel finding: VEGF levels were significantly elevated in samples exhibiting high CD47 expression compared to those with low CD47 levels (Fig. 4B). This observation was further supported by the immunohistochemistry results obtained from our comprehensive collection of 89 gastric cancer specimens (Fig. 4C). The results of this study indicate that the combination of CD47 and VEGF blockade may be a viable approach for treating gastric cancer. Furthermore, analysis of gene expression data from the GEPIA database supports the positive association between VEGF and CD47 in gastric cancer tissues (Fig. 4D). These findings provide evidence that CD47 expression is positively linked to VEGF expression in gastric cancer samples. Consequently, we propose that targeting tumor neovascularization could potentially impact the efficacy of anti-CD47 therapy.

### CD47 blockade combined with anti-angiogenetic therapy elicited enhanced anti-tumor effect

**Fig. 5:**
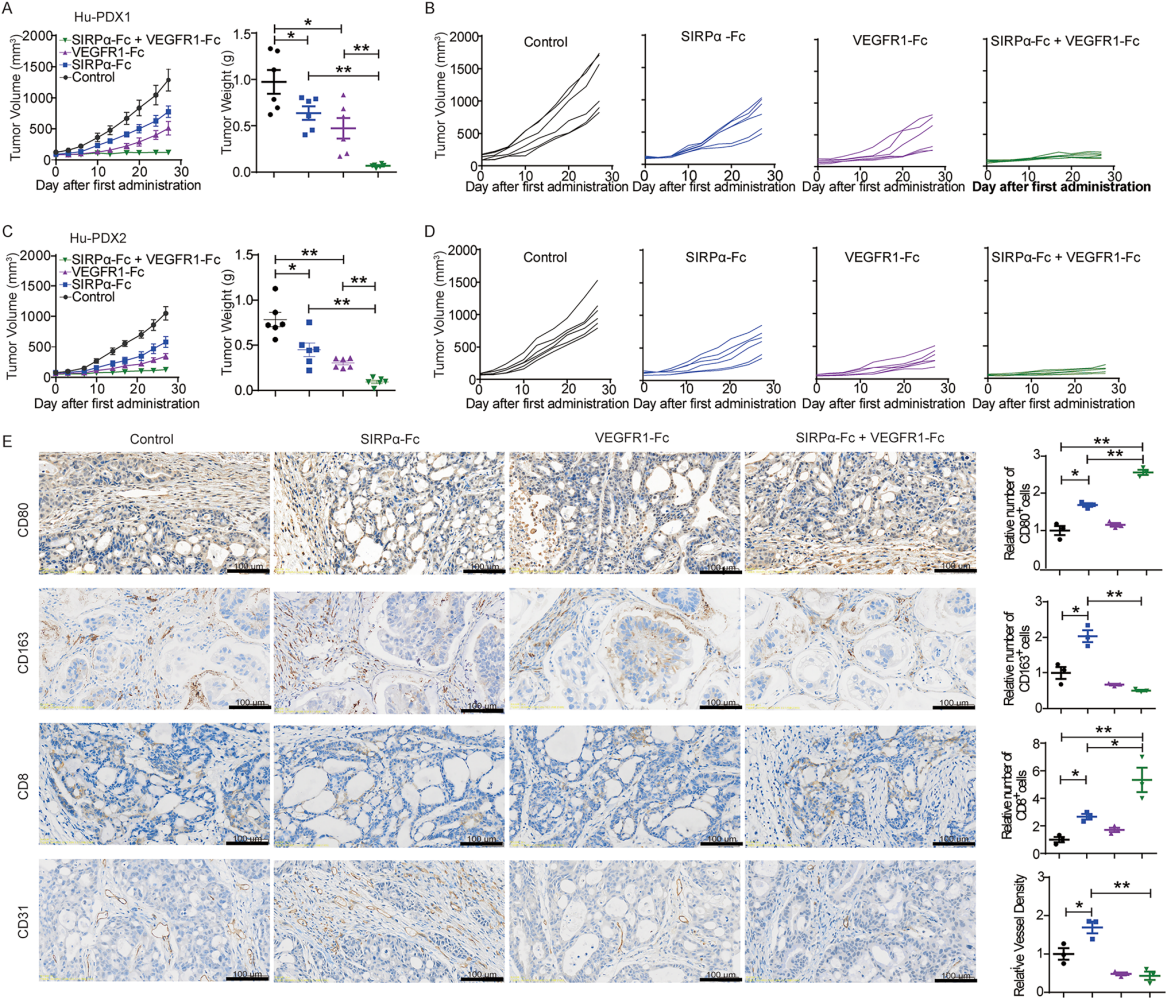
CD47 blockade combined with antiangiogenetic therapy elicited enhanced anti-tumor effect in Hu-PDX models of gastric cancer. **A** and** B** In the Hu-PDX1 model, tumor-bearing mice were treated with SIRPα-Fc and/or VEGFR1-Fc for 4 weeks, tumor volume and tumor weight were presented as mean ± SD. Each line represented the value of the tumor volume of a single mouse. **C** and** D** In the Hu-PDX2 model, tumor volume and tumor weight were presented after the same treatment in the Hu-PDX1 model. Each line represented the value of the tumor volume of a single mouse. (*n* = 6 per group, *P < 0.05, **P < 0.01). **E** Representative photographs of immunohistochemical staining for CD80, CD163, CD8, and CD31 of tumor tissue sections and the number of CD80^+^, CD163^+^, and CD8^+^ cells and the relative vessel density in each group were normalized to the control group. The value of control was set to 1.0. (*P < 0.05, **P < 0.01)

Subsequently, an investigation was conducted to determine whether the inhibition of VEGF could augment the effectiveness of CD47 blockade in gastric cancer. The results depicted demonstrate that the combination of SIRPα-Fc and VEGFR1-Fc resulted in the complete suppression of tumor growth in the Hu-PDX1 model (Fig. 5A, B). The tumor weights in the control group, SIRPα-Fc group, VEGFR1-Fc group, and SIRPα-Fc + VEGFR1-Fc group were recorded as 962.36 ± 171.10 mg, 514.50 ± 83.86 mg, 381.40 ± 95.65 mg, and 68.47 ± 7.53 mg, respectively. Similar findings were observed in the Hu-PDX2 model (Fig. 5C, D). Moreover, an examination was conducted on the presence of macrophages, CD8^+^ T cells, and microvessel density within the TME. Immunohistochemical analysis revealed that the combination of anti-angiogenic therapy and CD47 blockade resulted in a reduction of tumor angiogenic vasculature and infiltrated M2-like macrophages, in comparison to the use of anti-CD47 therapy alone. Furthermore, the combined treatment exhibited the greatest ability to induce infiltration of CD8^+^ T cells when compared to all other groups (Fig. 5E). The data collectively indicate that the concurrent utilization of angiogenetic axis blockade and CD47/SIRPα axis inhibition can effectively stimulate both the innate and adaptive immune systems, resulting in a synergistic anti-tumor response.

### Dual blocking CD47 and VEGF elicited synergetic anti-tumor effect and prevented gastric cancer recurrence

**Fig. 6:**
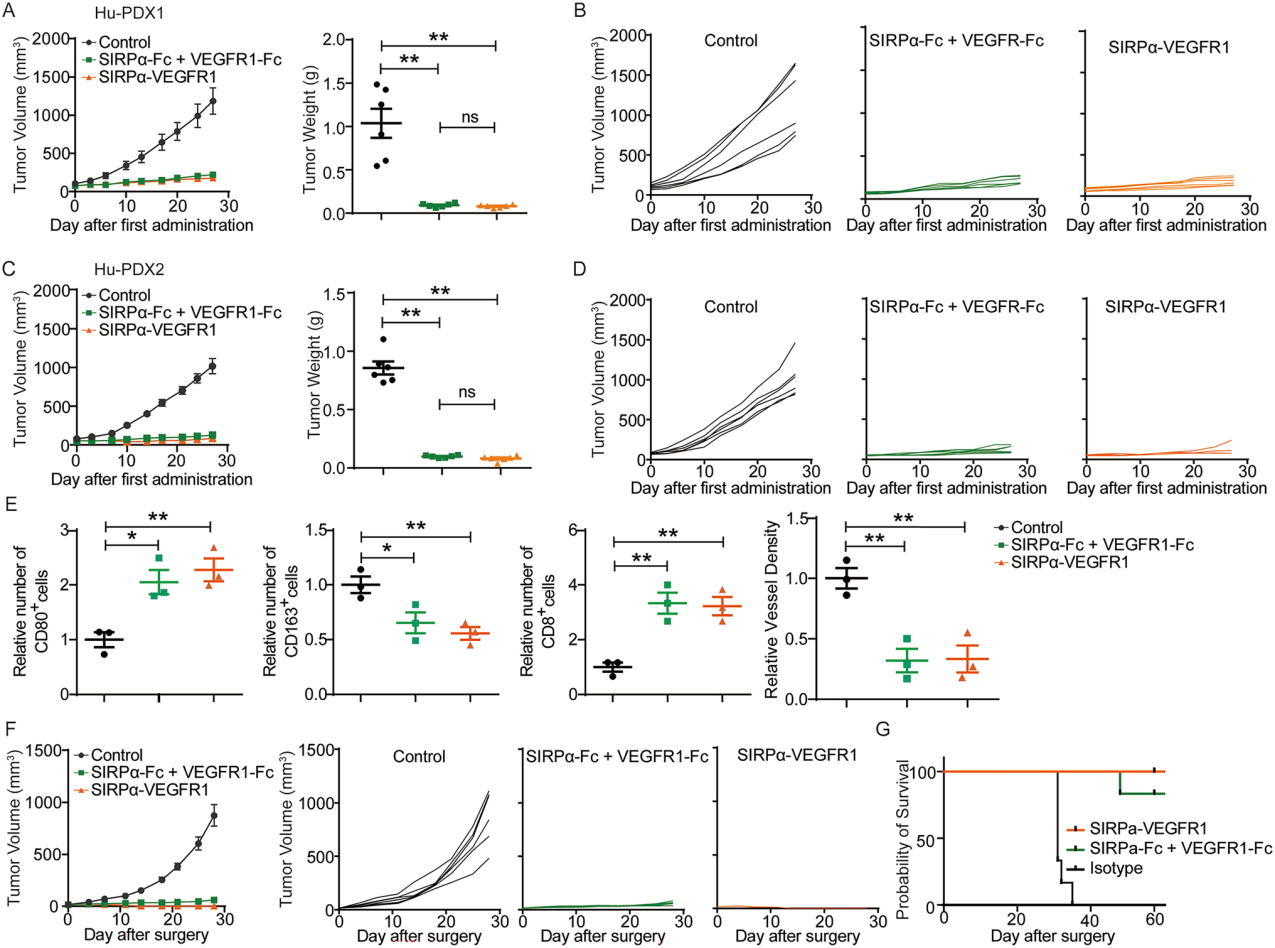
Bispecific fusion protein SIRPα-VEGFR1 elicited synergetic antitumor effect and prevented gastric cancer recurrence. **A** and** B** In the Hu-PDX1 model, tumor volume and tumor weight were measured and the data was presented as mean ± SD after treatment with SIRPα-Fc plus VEGFR1-Fc, and SIRPα-VEGFR1 for 4 weeks. Each line represented the value of the tumor volume of a single mouse. **C** and** D** In the Hu-PDX2 model, tumor volume and tumor weight were measured after the same treatment in the Hu-PDX1 model. **E** The number of CD80^+^, CD163^+^, and CD8^+^ cells and the relative vessel density in each group were normalized to the control group. The value of control was set to 1.0. (*P < 0.05, **P < 0.01). **F** In the humanized tumor recurrence model, mice were treated with control, SIRPα-Fc + VEGFR1-Fc, SIRPα-VEGFR1 for 2 weeks, and tumor volume was measured. Each line represented the value of the tumor volume of a single mouse. **G** Survival curves for different treatment groups. (n = 6 per group, *P < 0.05, **P < 0.01)

The evaluation of the anti-tumor effects of SIRPα-VEGFR1, a bispecific fusion protein that concurrently inhibits CD47 and VEGF, demonstrated significant tumor regression in the Hu-PDX1 model, comparable to the observed effects in the SIRPα-Fc + VEGFR1-Fc combination group (Fig. 6A, B). This underscores the significance of employing bispecific fusion proteins as a method to enhance the targeting efficacy in vivo and improve patient adherence. Following a treatment duration of 27 days, the tumor weights in the control group, SIRPα-Fc + VEGFR1-Fc group, and SIRPα-VEGFR1 group were measured at 905.30 ± 167.60 mg, 72.82 ± 8.65 mg, and 83.30 ± 11.71 mg, respectively. In the Hu-PDX2 model, comparable outcomes were observed in terms of tumor weights among the control group (826.30 ± 71.52 mg), the combinational administration group (98.33 ± 4.379 mg), and the bispecific fusion protein group (80.78 ± 9.63 mg) (Fig. 6C, D). Analysis of the TME revealed that the impact of SIRPα-VEGFR1 on macrophages, CD8^+^ T cells, and microvessel density is akin to that of the combination therapy (Fig. 6E). In the clinical setting, the occurrence of recurrence plays a significant role in the high mortality rates observed in patients with gastric cancer. Consequently, we established a post-surgical gastric cancer model to assess the efficacy of SIRPα-VEGFR1 in preventing recurrence. The surgical procedure resulted in incomplete removal of the tumor. Subsequently, the mice were administered injections of IgG1-Fc, SIRPα-Fc + VEGFR1-Fc, or SIRPα-VEGFR1. In comparison to the control group, the co-targeting of CD47 and VEGF demonstrated effective inhibition of tumor recurrence (Fig. 6F). Furthermore, all mice receiving SIRPα-VEGFR1 treatment remained alive for a period of 60 days following the surgery (Fig. 6G). In summary, the simultaneous inhibition of CD47 and VEGF using SIRPα-VEGFR1 successfully stimulated the immune response of the host against cancer recurrence, leading to a significant extension in overall survival.

## Discussion

In recent years, with more understanding of the molecular characteristics and heterogeneity of gastric cancer, great progress has been made in the treatment of gastric cancer. Targeted therapy and immunotherapy have changed the direction of the treatment of advance gastric cancer. Trastuzumab (anti-HER2 antibody) showed benefit in patients with HER2-positive tumors enrolled in the TOGA phase III trial in 2010 and is recommended in the first line in HER2-positive gastric cancer [34]. ICIs, especially with blocking PD-1/PD-L1 axis showed reliable and consistent efficacy in the refractory gastric cancer. In the phase III CheckMate-649 trial, Nivolumab showed benefit in combination with chemotherapy (oxaliplatin and fluoropyrimidines) over chemotherapy alone [35]. Pembrolizumab monotherapy demonstrated the clinical benefit in patients with previously treated unresectable or metastatic microsatellite instability-high (MSI-H) cancer in the phase II KEYNOTE-158 trial [36]. In HER2-positive and PD-L1-positive gastric cancer, pembrolizumab combined with first-line trastuzumab and chemotherapy significantly improved progression-free survival [37]. The combination of anti-angiogenic and ICI therapies was also studied in a phase Ib trial and showed encouraging anti-tumor effect in gastric cancer [38]. CD47 has also been proven to be a potential target for cancer immunotherapy [39]. There is mounting evidence that tumor cells increase the expression of CD47 to evade detection by the innate immune system [4]. Blocking CD47 not only directly enhances the ability of macrophages to engulf tumor cells, but also activates effector T cells and strengthens the adaptive immune response by facilitating the presentation of tumor antigens on macrophages [40–42]. CD47 blocking agents, including anti-CD47 antibody, anti-SIRPα antibody, and SIRPα-Fc fusion protein, have demonstrated significant efficacy in suppressing the proliferation of diverse tumor types, encompassing colon cancer, breast cancer, pancreatic neuroendocrine tumors, ovarian cancer, leiomyosarcoma, glioblastoma, and small-cell lung cancer [6, 13, 43–45]. Nevertheless, the efficacy of CD47 as a viable therapeutic target for gastric cancer remains uncertain owing to contradictory outcomes reported in prior research. For instance, Yoshida et al. have demonstrated that CD47 serves as an autonomous negative prognostic indicator in gastric cancer [46]. In contrast, an alternative investigation has revealed no statistically substantial variation in CD47 mRNA levels between primary gastric cancer and healthy tissues, thereby concluding that CD47 in primary gastric tumors does not exhibit any correlation with clinicopathological factors or prognosis [47]. In this study, clinical specimens were obtained from a cohort of 89 patients diagnosed with gastric cancer, and subsequent analysis was conducted to assess the expression of CD47 in both tumor tissues and corresponding normal tissues. The findings of our investigation substantiated the overexpression of CD47 in gastric cancer, establishing a significant association with unfavorable prognosis. Furthermore, we proceeded to investigate the therapeutic potential of anti-CD47 treatment in gastric cancer, revealing that the blockade of CD47 effectively impeded tumor growth by activating macrophage-mediated innate and adaptive immune responses.

The application of immunotherapy in cancer treatment has revealed multiple instances of both intrinsic and acquired resistance [48]. Despite the demonstrated anti-tumor effects of immune checkpoint inhibitors (ICIs) in various cancer types, eradicating tumor cells remains challenging, including in the case of CD47 blockade therapy [49–51]. Consequently, several studies have been conducted to explore novel strategies aimed at achieving a more potent antitumor effect. For instance, the combination of anti-GD2 and anti-CD47 has been found to synergistically facilitate the eradication of neuroblastoma by altering both pro- and anti-phagocytic signals within the tumor microenvironment [52]. The efficacy of immunotherapy against melanoma and colorectal cancer was enhanced by the simultaneous blocking of CD47 and PD-L1 using bispecific antibodies, which activated both innate and adaptive immune responses [53–55]. The combination of autophagy inhibitors with anti-CD47 therapy further improved tumoricidal activity by regulating catabolic pathways and cellular homeostasis [15, 56]. More recently, targeting CD47 by SIRPα-Fc fusion protein or monoclonal antibody combined with azacitidine showed reliable efficacy and safety in some malignant hematological tumors such as myelodysplastic syndrome, acute myeloid leukemia and chronic myelomonocytic leukemia in clinical trials [57–60]. In our study, we observed a significant presence of microvessels in gastric cancer following anti-CD47 treatment, leading us to discover that tumor angiogenesis plays a crucial role in limiting the effectiveness of anti-CD47 therapy for the first time.

Tumor neovascularization hinders the infiltration of immune cells that react to tumors and fosters an immunosuppressive TME that enables tumors to resist immunotherapy. In the majority of cancer cases, VEGF promotes neovascularization, impairs the interaction between endothelial cells and leukocytes, and limits the infiltration of immune cells into the TME by reducing adhesion molecules. Simultaneously, elevated levels of VEGF and angiogenesis in tumors impede the cytotoxic activity of CD8^+^ T cells and enhance the presence of immunosuppressive cells, such as regulatory T cells (Treg cells) and M2-like macrophages [61]. In the current investigation, it was observed that administration of anti-CD47 treatment resulted in an elevation in microvessel density within the tumor [62]. This finding is significant as it aligns with previous research indicating that anti-VEGF agents can normalize tumor vasculature and transform the immunosuppressive TME into an immunosupportive one. Consequently, this alteration in the TME has been shown to enhance the efficacy of PD-1/PD-L1 inhibitors in various cancers, including gastric cancer. Furthermore, the combination of anti-VEGF/VEGFR and PD-1/PD-L1 inhibitors has been granted FDA approval for the treatment of hepatocellular carcinoma and renal cell carcinoma [63–65]. It is noteworthy that the blockade of CD47 has been shown to alter the phenotype of macrophages toward the M1 subtype, which has anti-tumorigenic properties, as demonstrated in previous studies [66]. However, our study also observed an increase in the presence of pro-tumorigenic M2 macrophages in the tumor microenvironment, potentially attributed to the influence of tumor angiogenesis on the recruitment and proliferation of M2 macrophages. Notably, our study also revealed a positive correlation between VEGF expression and CD47 in gastric cancer tissues, suggesting the potential efficacy of combining anti-CD47 and anti-angiogenesis therapies for the treatment of gastric cancer in clinical settings. To investigate the impact of tumor neovascularization on tumor growth and the potential interference with the anti-tumor effect of CD47 blockade within the tumor immune microenvironment, Hu-PDX models were subjected to the administration of SIRPα-Fc and VEGFR1-Fc, aiming to achieve a simultaneous blockade of CD47 and tumor angiogenesis. The findings revealed that this combined therapeutic approach resulted in a synergistic anti-tumor effect against gastric cancer, characterized by a reduction in microvessel density and M2 macrophages. The bispecific fusion protein SIRPα-VEGFR1 was employed to concurrently target CD47 and VEGF, resulting in significant suppression of tumor growth in the Hu-PDX model. Furthermore, SIRPα-VEGFR1 demonstrated a sustained immune response, effectively preventing the recurrence of gastric cancer and extending overall survival in a recurrence model.

In summary, our research has validated the elevated expression of CD47 in human gastric cancer and its significant association with unfavorable prognosis. The utilization of SIRPα-Fc in anti-CD47 therapy has demonstrated remarkable efficacy in Hu-PDX models of gastric cancer. However, it is worth noting that the administration of anti-CD47 treatment led to an escalation in microvessel density within the tumor, potentially fostering an immunosuppressive microenvironment and constraining the effectiveness of CD47 blockade. Additionally, there exists a positive correlation between VEGF expression and CD47 expression in gastric cancer. The concurrent administration of anti-CD47 and anti-VEGF therapies, along with the utilization of a bispecific fusion protein, yielded notable enhancements in the tumor immune microenvironment and a substantial augmentation of the anti-tumor response. These findings strongly indicate that CD47 holds promise as a viable therapeutic target for gastric cancer, and underscore the considerable advantages of combining anti-CD47 treatment with anti-angiogenic therapy in the context of gastric cancer treatment.

### Supplementary Information

Below is the link to the electronic supplementary material.

Supplementary file1 (XLSX 11 KB)

## Data Availability

The survival analysis data between relapse-free survival and stomach adenocarcinoma are available online through the Kaplan–Meier plotter based on RNA-seq. The correlation between CD47 and VEGF data is available online through Correlation Analysis in GEPIA based on the TCGA STAD tumor database. All other data that support the findings of this study are available from the corresponding author upon reasonable request.

## Abbreviations

AJCC: American joint committee on cancer
ANOVA: Analysis of variance
BSA: Bovine serum albumin
CFDA SE: Carboxyfluorescein diacetate succinimidyl ester
GEPIA: Gene expression profiling interactive analysis
GM-CSF: Granulocyte–macrophage colony-stimulating factor
Hu-PDX: Patient-derived xenograft
Hu-PDXs: Humanized patient-derived xenografts
ICIs: Immune checkpoint inhibitors
IHC: Immunohistochemical
KM-plotter: Kaplan–Meier plotter
NCG mice: A severely immunodeficient strain obtained by knocking out the Prkdc and Il2rg genes in NOD/ShiltJGpt mice
PBMCs: Peripheral blood mononuclear cells
PDC: Patient-derived cell
SIRPα: Signal regulatory protein alpha
SIRPα: Signal regulatory protein α
TME: Tumor microenvironment
VEGF: Vascular endothelial growth factor

## References

[1] Sung H, Ferlay J, Siegel RL, Laversanne M, Soerjomataram I, Jemal A, Bray F (2021). Global cancer statistics 2020: GLOBOCAN estimates of incidence and mortality worldwide for 36 cancers in 185 countries. CA Cancer J Clin.

[2] Smyth EC, Nilsson M, Grabsch HI, van Grieken NC, Lordick F (2020). Gastric cancer. Lancet.

[3] Körfer J, Lordick F, Hacker UT (2021). Molecular targets for gastric cancer treatment and future perspectives from a clinical and translational point of view. Cancers.

[4] Jaiswal S, Jamieson CH, Pang WW, Park CY, Chao MP, Majeti R, Traver D, van Rooijen N, Weissman IL (2009). CD47 is upregulated on circulating hematopoietic stem cells and leukemia cells to avoid phagocytosis. Cell.

[5] Chao MP, Alizadeh AA, Tang C, Jan M, Weissman-Tsukamoto R, Zhao F, Park CY, Weissman IL, Majeti R (2011). Therapeutic antibody targeting of CD47 eliminates human acute lymphoblastic leukemia. Cancer Res.

[6] Willingham SB, Volkmer JP, Gentles AJ (2012). The CD47-signal regulatory protein alpha (SIRPa) interaction is a therapeutic target for human solid tumors. Proc Natl Acad Sci U S A.

[7] Li Y, Zhang M, Wang X, Liu W, Wang H, Yang YG (2020). Vaccination with CD47 deficient tumor cells elicits an antitumor immune response in mice. Nat Commun.

[8] Vonderheide RH (2015). CD47 blockade as another immune checkpoint therapy for cancer. Nat Med.

[9] Liu X, Pu Y, Cron K (2015). CD47 blockade triggers T cell-mediated destruction of immunogenic tumors. Nat Med.

[10] Majeti R, Chao MP, Alizadeh AA, Pang WW, Jaiswal S, Gibbs KD, van Rooijen N, Weissman IL (2009). CD47 is an adverse prognostic factor and therapeutic antibody target on human acute myeloid leukemia stem cells. Cell.

[11] Barrera L, Montes-Servín E, Hernandez-Martinez JM, García-Vicente M, Montes-Servín E, Herrera-Martínez M, Crispín JC, Borbolla-Escoboza JR, Arrieta O (2017). CD47 overexpression is associated with decreased neutrophil apoptosis/phagocytosis and poor prognosis in non-small-cell lung cancer patients. Br J Cancer.

[12] Fu W, Li J, Zhang W, Li P (2017). High expression of CD47 predicts adverse prognosis in Chinese patients and suppresses immune response in melanoma. Biomed Pharmacother.

[13] Weiskopf K, Jahchan NS, Schnorr PJ (2016). CD47-blocking immunotherapies stimulate macrophage-mediated destruction of small-cell lung cancer. J Clin Invest.

[14] Schürch CM, Roelli MA, Forster S (2019). Targeting CD47 in anaplastic thyroid carcinoma enhances tumor phagocytosis by macrophages and is a promising therapeutic strategy. Thyroid.

[15] Zhang X, Chen W, Fan J (2018). Disrupting CD47-SIRPα axis alone or combined with autophagy depletion for the therapy of glioblastoma. Carcinogenesis.

[16] Khan KA, Kerbel RS (2018). Improving immunotherapy outcomes with anti-angiogenic treatments and vice versa. Nat Rev Clin Oncol.

[17] Chen DS, Hurwitz H (2018). Combinations of bevacizumab with cancer immunotherapy. Cancer J.

[18] Ohm JE, Gabrilovich DI, Sempowski GD, Kisseleva E, Parman KS, Nadaf S, Carbone DP (2003). VEGF inhibits T-cell development and may contribute to tumor-induced immune suppression. Blood.

[19] Ferrara N (2010). Binding to the extracellular matrix and proteolytic processing: two key mechanisms regulating vascular endothelial growth factor action. Mol Biol Cell.

[20] Ferrara N (2010). Vascular endothelial growth factor and age-related macular degeneration: from basic science to therapy. Nat Med.

[21] Jain RK (2003). Molecular regulation of vessel maturation. Nat Med.

[22] Huang Y, Goel S, Duda DG, Fukumura D, Jain RK (2013). Vascular normalization as an emerging strategy to enhance cancer immunotherapy. Cancer Res.

[23] Huang Y, Yuan J, Righi E (2012). Vascular normalizing doses of antiangiogenic treatment reprogram the immunosuppressive tumor microenvironment and enhance immunotherapy. Proc Natl Acad Sci USA.

[24] Jain RK (2005). Normalization of tumor vasculature: an emerging concept in antiangiogenic therapy. Science.

[25] Ziogas AC, Gavalas NG, Tsiatas M (2012). VEGF directly suppresses activation of T cells from ovarian cancer patients and healthy individuals via VEGF receptor Type 2. Int J Cancer.

[26] Gabrilovich DI, Chen HL, Girgis KR, Cunningham HT, Meny GM, Nadaf S, Kavanaugh D, Carbone DP (1996). Production of vascular endothelial growth factor by human tumors inhibits the functional maturation of dendritic cells. Nat Med.

[27] Maenhout SK, Thielemans K, Aerts JL (2014). Location, location, location: functional and phenotypic heterogeneity between tumor-infiltrating and non-infiltrating myeloid-derived suppressor cells. Oncoimmunology.

[28] Linde N, Lederle W, Depner S, van Rooijen N, Gutschalk CM, Mueller MM (2012). Vascular endothelial growth factor-induced skin carcinogenesis depends on recruitment and alternative activation of macrophages. J Pathol.

[29] Allen E, Jabouille A, Rivera LB (2017). Combined antiangiogenic and anti-PD-L1 therapy stimulates tumor immunity through HEV formation. Sci Transl Med.

[30] Liguigli W, Tomasello G, Toppo L, Ratti M, Passalacqua R (2014). Ramucirumab for metastatic gastric or gastroesophageal junction cancer: results and implications of the REGARD trial. Future Oncol.

[31] Jin Z, Yoon HH (2017). Antiangiogenic therapy in gastroesophageal cancer. Hematol Oncol Clin North Am.

[32] Yu J, Li S, Chen D (2020). Crystal structure of human cd47 in complex with engineered SIRPα.D1(N80A). Molecules..

[33] Xu C, Zhu M, Wang Q (2023). TROP2-directed nanobody-drug conjugate elicited potent antitumor effect in pancreatic cancer. J Nanobiotechnology.

[34] Bang YJ, Van Cutsem E, Feyereislova A (2010). Trastuzumab in combination with chemotherapy versus chemotherapy alone for treatment of HER2-positive advanced gastric or gastro-oesophageal junction cancer (ToGA): a phase 3, open-label, randomised controlled trial. Lancet.

[35] Janjigian YY, Shitara K, Moehler M (2021). First-line nivolumab plus chemotherapy versus chemotherapy alone for advanced gastric, gastro-oesophageal junction, and oesophageal adenocarcinoma (CheckMate 649): a randomised, open-label, phase 3 trial. Lancet.

[36] Marabelle A, Le DT, Ascierto PA (2020). Efficacy of pembrolizumab in patients with noncolorectal high microsatellite instability/mismatch repair-deficient cancer: results from the phase II KEYNOTE-158 study. J Clin Oncol.

[37] Janjigian YY, Kawazoe A, Bai Y (2023). Pembrolizumab plus trastuzumab and chemotherapy for HER2-positive gastric or gastro-oesophageal junction adenocarcinoma: interim analyses from the phase 3 KEYNOTE-811 randomised placebo-controlled trial. Lancet.

[38] Fukuoka S, Hara H, Takahashi N (2020). Regorafenib plus nivolumab in patients with advanced gastric or colorectal cancer: an open-label, dose-escalation, and dose-expansion phase Ib trial (REGONIVO, EPOC1603). J Clin Oncol.

[39] Jiang Z, Sun H, Yu J, Tian W, Song Y (2021). Targeting CD47 for cancer immunotherapy. J Hematol Oncol.

[40] Li Y, Liu J, Chen W, Wang W, Yang F, Liu X, Sheng Y, Du K, He M, Lyu X, Li H, Zhao L, Wei Z, Wang F, Zheng S, Sui J (2023). A pH-dependent anti-CD47 antibody that selectively targets solid tumors and improves therapeutic efficacy and safety. J Hematol Oncol.

[41] Tseng D, Volkmer JP, Willingham SB (2013). Anti-CD47 antibody-mediated phagocytosis of cancer by macrophages primes an effective antitumor T-cell response. Proc Natl Acad Sci U S A.

[42] von Roemeling CA, Wang Y, Qie Y (2020). Therapeutic modulation of phagocytosis in glioblastoma can activate both innate and adaptive antitumour immunity. Nat Commun.

[43] Yu J, Li S, Chen D, Liu D, Guo H, Yang C, Zhang W, Zhang L, Zhao G, Tu X, Peng L, Liu S, Bai X, Song Y, Jiang Z, Zhang R, Tian W (2022). SIRPα-Fc fusion protein IMM01 exhibits dual anti-tumor activities by targeting CD47/SIRPα signal pathway via blocking the “don't eat me” signal and activating the “eat me” signal. J Hematol Oncol.

[44] Edris B, Weiskopf K, Volkmer AK (2012). Antibody therapy targeting the CD47 protein is effective in a model of aggressive metastatic leiomyosarcoma. Proc Natl Acad Sci U S A.

[45] Krampitz GW, George BM, Willingham SB (2016). Identification of tumorigenic cells and therapeutic targets in pancreatic neuroendocrine tumors. Proc Natl Acad Sci U S A.

[46] Yoshida K, Tsujimoto H, Matsumura K (2015). CD47 is an adverse prognostic factor and a therapeutic target in gastric cancer. Cancer Med.

[47] Sudo T, Takahashi Y, Sawada G, Uchi R, Mimori K, Akagi Y (2017). Significance of CD47 expression in gastric cancer. Oncol Lett.

[48] Morad G (2022). Hallmarks of response, resistance, and toxicity to oncol lettmmune checkpoint blockade. Cell.

[49] Chao MP, Alizadeh AA, Tang C (2010). Anti-CD47 antibody synergizes with rituximab to promote phagocytosis and eradicate non-Hodgkin lymphoma. Cell.

[50] Bagchi S, Yuan R, Engleman EG (2021). Immune checkpoint inhibitors for the treatment of cancer: clinical impact and mechanisms of response and resistance. Annu Rev Pathol.

[51] Lu Z, Peng Z, Liu C, Wang Z, Wang Y, Jiao X, Li J, Shen L (2020). Current status and future perspective of immunotherapy in gastrointestinal cancers. Innovation (Camb).

[52] Theruvath J, Menard M, Smith BAH (2022). Anti-GD2 synergizes with CD47 blockade to mediate tumor eradication. Nat Med.

[53] Wang Y, Ni H, Zhou S (2021). Tumor-selective blockade of CD47 signaling with a CD47/PD-L1 bispecific antibody for enhanced anti-tumor activity and limited toxicity. Cancer Immunol Immunother.

[54] Chen SH, Dominik PK, Stanfield J (2021). Dual checkpoint blockade of CD47 and PD-L1 using an affinity-tuned bispecific antibody maximizes antitumor immunity. J Immunother Cancer.

[55] Liu B, Guo H, Xu J (2018). Elimination of tumor by CD47/PD-L1 dual-targeting fusion protein that engages innate and adaptive immune responses. mAbs.

[56] Zhang X, Fan J, Wang S (2017). Targeting CD47 and autophagy elicited enhanced antitumor effects in non-small cell lung cancer. Cancer Immunol Res.

[57] Tong H, Yang W, Li J et al. (2023) Preliminary results of a phase 2 Study of IMM01 combined with azacitidine (AZA) As the first-line treatment in adult patients with chronic myelomonocytic leukemia (CMML). ASH. https://ash.confex.com/ash/2023/webprogram/Paper181501.html Accessed 9 December 2023

[58] Yang W, Yan X, Guo R et al. (2023) Preliminary results of a phase 2 study of IMM01 combined with azacitidine (aza) as the first-line treatment in adult patients with higher risk myelodysplastic syndromes (MDS). ASH. https://ash.confex.com/ash/2023/webprogram/Paper174420.html. Accessed 9 December 2023

[59] Wang H, Teng Q, Li Z, Liu H, Wang Z, Li B, Xia Y, Jin J (2023) A phase 1b study evaluating the safety and efficacy of AK117 (anti-CD47 monoclonal antibody) in combination with azacitidine in patients with treatment-naïve acute myeloid leukemia. ASH. https://ash.confex.com/ash/2023/webprogram/Paper180618.html Accessed 11 December 2023

[60] Miao M, Wu D, Jiang Z, Jiang S, Li F, Liang Y, Wang Z, Li B, Xia Y Tong H (2023) AK117 (anti-CD47 monoclonal antibody) in combination with Azacitidine for newly diagnosed higher risk myelodysplastic syndrome (HR-MDS): AK117–103 Phase 1b Results. ASH. https://ash.confex.com/ash/2023/webprogram/Paper179099.html. Accessed 9 December 2023

[61] Voron T, Colussi O, Marcheteau E (2015). VEGF-A modulates expression of inhibitory checkpoints on CD8+ T cells in tumors. J Exp Med.

[62] Jain RK (2001). Normalizing tumor vasculature with anti-angiogenic therapy: a new paradigm for combination therapy. Nat Med.

[63] Saeed A, Park R, Sun W (2021). The integration of immune checkpoint inhibitors with VEGF targeted agents in advanced gastric and gastroesophageal adenocarcinoma: a review on the rationale and results of early phase trials. J Hematol Oncol.

[64] Shigeta K, Datta M, Hato T (2020). Dual programmed death receptor-1 and vascular endothelial growth factor receptor-2 blockade promotes vascular normalization and enhances antitumor immune responses in hepatocellular carcinoma. Hepatology.

[65] Meder L, Schuldt P, Thelen M (2018). Combined VEGF and PD-L1 blockade displays synergistic treatment effects in an autochthonous mouse model of small cell lung cancer. Cancer Res.

[66] Zhang M, Hutter G, Kahn SA (2016). Anti-CD47 treatment stimulates phagocytosis of glioblastoma by M1 and M2 polarized macrophages and promotes M1 polarized macrophages in vivo. PLoS ONE.

